# Aerobic Exercise Increases Meteorin-Like Protein in Muscle and Adipose Tissue of Chronic High-Fat Diet-Induced Obese Mice

**DOI:** 10.1155/2018/6283932

**Published:** 2018-04-30

**Authors:** Ju Yong Bae

**Affiliations:** Laboratory of Exercise Biochemistry, Department of Physical Education, College of Arts and Physical Education, Dong-A University, 37 Nakdong-daero 550 beon-gil, Hadan-dong, Saha-gu, Busan 604-714, Republic of Korea

## Abstract

Upregulated meteorin-like (Metrnl) protein in peripheral tissues because of exercise-induced increases in intramuscular Metrnl may effectively alleviate obesity by improving metabolism in whole-body tissues. The objective was to analyse the effects of regular treadmill exercise on Metrnl levels in muscle and peripheral tissues of chronic high-fat diet- (HFD-) induced obese mice. Forty-eight-week-old male C57BL/6 mice were first divided equally into normal-diet (CO) and high-fat diet (HF) groups. Following 16 weeks of a HFD, each group was again split equally into control (CO, HF) and training groups (COT, HFT). The HFT group expressed significantly higher phospho-AMP-activated protein kinase (AMPK), AMPK activity, and peroxisome proliferator-activated receptor gamma coactivator-1*α* (PGC-1*α*) in muscle tissue than the HF group (*p* < 0.05). Similar to muscle energy sensing network protein levels, the HFT group also expressed significantly higher muscle, plasma, and adipose tissue Metrnl (*p* < 0.05). Moreover, regular exercise increased acyl-CoA oxidase 1 (ACOX-1) and monoglyceride lipase (MGL) expression in adipose tissue (*p* < 0.05) and significantly decreased abdominal fat mass (*p* < 0.05). This study suggests that exercise-induced muscle Metrnl effectively reduces fat accumulation through the increase of Metrnl in adipose tissue, which may be a therapeutic target for chronic obesity.

## 1. Introduction

The risks of a continuous high-fat diet (HFD) and obesity are well established. However, the prevalence of obesity is increasing globally [1]. Therefore, appropriate solutions for a reduction in obesity prevalence are required. Ingested fat is primarily stored in the form of triglycerides (TGs) [2], but chronic nutrition in excess of the storage capacity of adipose tissue causes TGs to be deposited in the internal organs, leading to steatosis and organ dysfunction [3]. Obesity resulting from a HFD is associated with significant metabolic disturbance and may induce pathology in various organs [4–6].

Exercise is one of the most effective treatments to alleviate obesity. Exercise through repeated muscle contraction and relaxation promotes the release of metabolism-related proteins in skeletal muscle. Both aerobic and resistance exercises improve insulin sensitivity [7] by improving insulin action and glucose uptake [8, 9]. Recent studies report that regular aerobic exercise improves leptin [6] and insulin resistance [10] in peripheral tissues. Therefore, regular exercise may be an effective preventive and therapeutic treatment for metabolic disturbance in HFD-induced obesity through upregulation of metabolism-related proteins, not only in muscle, but also in peripheral tissues.

Meteorin-like (Metrnl) protein, also known as subfatin, is transcribed similarly to the meteorin protein [11]. When it was first described, the Metrnl protein was annotated as meteorin-like in public databases because neither its expression nor functions had been reported [12]. Recent studies on the function of Metrnl have focused specifically on its role as a myokine and cytokine. Previous study reported that adipocyte Metrnl controls insulin sensitivity through the peroxisome proliferator-activated receptor gamma (PPAR*γ*) pathway and suggested that adipocyte Metrnl is an inherent insulin sensitizer and may be a therapeutic target for insulin resistance [11]. Rao et al. suggested that upregulated peroxisome proliferator-activated receptor gamma coactivator-1*α* (PGC-1*α*), a major regulator that strongly induces mitochondrial biosynthesis, increases Metrnl in muscle tissue. Following this upregulation, Metrnl then transfers the positive effects of PGC-1*α* to other tissues [13].

Together, this evidence suggests that the upregulation of intramuscular Metrnl induced by regular aerobic exercise may be an effective pathway to alleviate obesity by improving the metabolism in whole-body tissues. Although Metrnl is thought to act positively in the case of chronic HFD-induced obesity, to our knowledge, there are no studies that have examined the changes in intramuscular and peripheral Metrnl levels induced by regular aerobic exercise. Therefore, the purpose of this study was to analyse the effects of regular aerobic exercise on Metrnl expression levels in muscle and peripheral tissues of chronic HFD-induced obese mice.

## 2. Materials and Methods

### 2.1. Animals

Forty-four-week-old male C57BL/6 mice were obtained from Samtako (Osan, Korea) and acclimatised for four weeks under standardised conditions in an animal facility (Dong-A University College of Medicine Animal Laboratory). Animal experimentation was approved by the Dong-A University Medical School Institutional Animal Care and Use Committee and all procedures were conducted in accordance with the Committee's Guidelines on Animal Experiments.

### 2.2. Obesity Induction

Following environmental adaptation, all animals were randomly assigned to a normal-diet + sedentary group (CO, *n* = 20) or a HFD + sedentary group (HF, *n* = 20). For 16 weeks, the HF group was fed a 60% fat diet (60% lipid, 20% carbohydrate, and 20% protein) to induce chronic HFD-induced obesity, whereas the CO was fed a standard diet. Thereafter, the CO group was divided into a CO group (*n* = 10) or a normal-diet + training group (COT, *n* = 10) and the HF group was divided into a HF group (*n* = 10) or a HFD + training group (HFT, *n* = 10). All animals had free access to tap water and body weight was measured each week at the same time.

### 2.3. Treadmill Training

A training protocol, which did not induce muscle damage in a previous study [2], was used. The COT and HFT groups performed aerobic exercise using an animal treadmill five times a week for eight weeks. The specific exercise program is shown in Table 1.

### 2.4. Blood and Tissue Sampling

Dissection was performed 48 hours after the last exercise to exclude the temporary exercise effect. After complete anaesthesia (ethyl ether), blood samples (1 mL) were obtained from the abdominal vena cava via syringes. Plasma was collected from heparinised blood by centrifugation for 15 min at 3000 rpm. The gastrocnemii of the right leg, liver, and abdominal visceral fat were excised and stored at −80°C until analysis.

### 2.5. Plasma Lipid Profiles and Metrnl Concentration

Plasma total cholesterol (TC) and TG concentrations were analysed using commercial kits (Asan Pharmaceutical, Korea) by the enzymatic colorimetric method. For determination of plasma high-density-lipoprotein-cholesterol (HDL-C) concentration, a commercial kit (Shinyang Diagnostics, Korea) was used and low-density-lipoprotein-cholesterol (LDL-C) was calculated with the following formula: LDL-C = TC − (HDL-C + TG/5) [14]. Plasma Metrnl protein concentration was determined with an Enzyme-Linked Immunosorbent Assay (ELISA) using the mouse Meteorin-like DuoSet ELISA Kit (DY6679, R&D System, USA) according to the manufacturer's instructions. A total of 100 *μ*l of sample (50 *μ*l sample and 50 *μ*l Reagent Diluent) were used to analyse the optical density, and the concentration was calculated by multiplying the sample value by the dilution factor.

### 2.6. Protein Analysis

Protein levels in tissues were assessed using western blot analysis. After washing with ice-cold phosphate-buffered saline (PBS), tissues were lysed in 200 *μ*l RIPA buffer and incubated at 4°C for 30 min. The tissues were then homogenised and centrifuged at 14,000 rpm for 30 min. Equal protein amounts were resolved using SDS-polyacrylamide gel electrophoresis on a 10 or 12% gel and transferred to a membrane. The membrane was blocked with 5% skim milk in PBS and subsequently incubated at 4°C overnight with primary antibodies (1 : 1000 dilution) against AMP-activated protein kinase (AMPK) (#2532, Cell Signaling Technology, USA), phospho-AMPK (p-AMPK) (#2535, Cell Signaling Technology), Sirtuin 1 (SIRT1) (#2028, Cell Signaling Technology), PGC-1*α* (ab54481, Abcam, USA), Meteorin-like (sc-168581, Santa Cruz, USA), acyl-CoA oxidase 1 (ACOX-1) (sc-98499, Santa Cruz), carnitine palmitoyl transferase I (CPT-1) (sc-98834, Santa Cruz), hormone-sensitive lipase (HSL) (sc-25843, Santa Cruz), and monoglyceride lipase (MGL) (sc-398942, Santa Cruz). The membrane was incubated with secondary antibody for one hour at room temperature. Immunostaining with antibodies was developed using an ECL solution (Amersham Pharmacia Biotech, USA) and detected with an ImageQuant LAS-4000 system (GE Healthcare, Sweden). Quantification of bands was performed using ImageJ 1.48q (NIH Imaging software, USA).

### 2.7. Statistical Analysis

All calculations were performed using the Statistical Package for Social Sciences version 22.0 (SPSS Inc., USA) and are presented as the mean ± standard error. Changes in body weight induced by the HFD were analysed using an independent *t*-test and ANOVA. One-way ANOVA and Duncan's post hoc analyses were performed for any intergroup differences observed. A statistical significance level was set at *p* < 0.05 for analysis.

## 3. Results

### 3.1. Changes in Body Weight, Fat Mass, and Liver Weight

**Figure 1:**
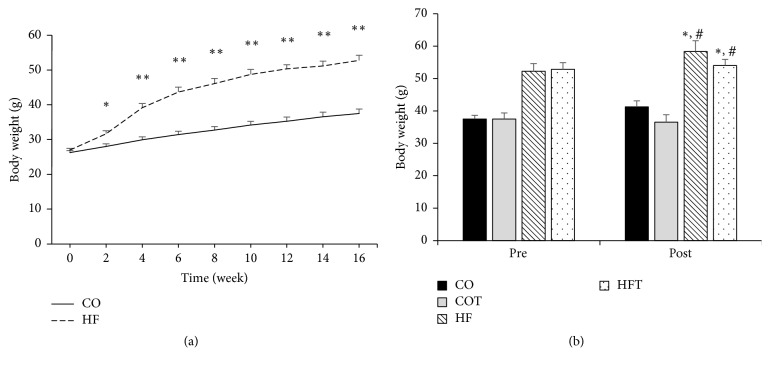
Changes in body weight. Changes in body weight during the obesity-induction period (a) and before and after the eight-week training programme (b). Data are expressed as mean ± SE. CO: normal-diet group, COT: normal-diet + training group, HF: high-fat diet group, and HFT: high-fat diet + training group. *∗* versus CO, *p* < 0.05; *∗∗* versus CO, *p* < 0.001; #; versus COT, *p* < 0.05.

**Figure 2:**
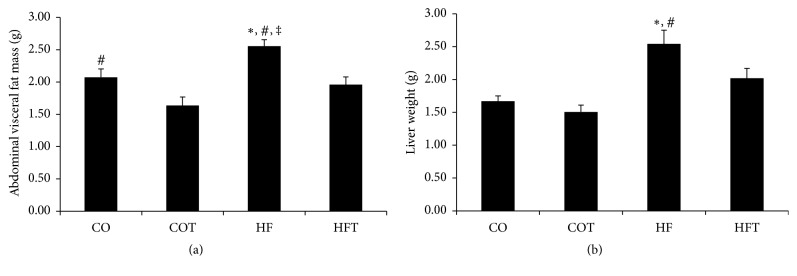
Changes in fat mass and liver weight following an eight-week training programme. Data are expressed as mean ± SE. CO: normal-diet group, COT: normal-diet + training group, HF: high-fat diet group, and HFT: high-fat diet + training group. *∗* versus CO, *p* < 0.05; # versus COT, *p* < 0.05; ‡ versus HFT, *p* < 0.05.

The HF group was fed a 60% HFD for 16 weeks before the eight-week treadmill training. There was a significant difference between the CO and HF groups after two weeks of the HFD (*p* < 0.05), and this difference increased gradually until the end of the obesity-induction period (*p* < 0.001). Following eight weeks of training, the body weights of the HF and HFT groups were significantly higher than those of the CO and COT groups (*p* < 0.05) (Figure 1). Abdominal fat mass and liver weight were reduced by the eight-week training. Abdominal visceral fat mass in the HF group was significantly higher than in all other groups (*p* < 0.05), and the fat mass in the HFT group was significantly lower than in the HF group (*p* < 0.05). Liver weight in the HF group was significantly higher than in the CO and COT groups (*p* < 0.05) (Figure 2).

### 3.2. Muscle Energy Sensing Network Proteins Level

**Figure 3:**
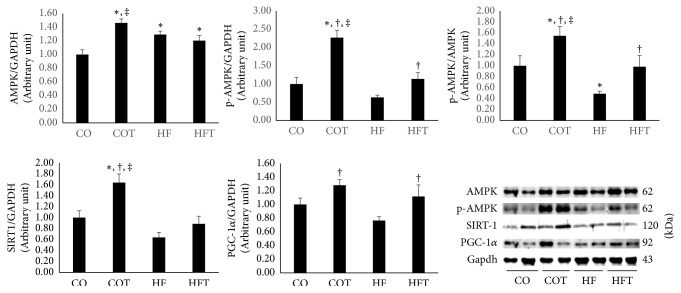
Intramuscular protein levels following an eight-week training programme. Data are expressed as mean ± SE. CO: normal-diet group, COT: normal-diet + training group, HF: high-fat diet group, and HFT: high-fat diet + training group. *∗* versus CO, *p* < 0.05; † versus HF, *p* < 0.05; ‡ versus HFT, *p* < 0.05.

Eight-week treadmill training increased muscle energy sensing network protein levels, while the chronic HFD decreased these levels (Figure 3). The level of AMPK was significantly higher in the COT group than in the CO and HFT groups. AMPK levels were also higher in the HF and HFT groups than in the CO group (*p* < 0.05). The level of p-AMPK was significantly higher in the COT group than in all other groups and significantly higher in the HFT group than in the HF group (*p* < 0.05). AMPK activity was also significantly higher in the COT group than in all other groups and significantly higher in the HFT group than in the HF group (*p* < 0.05). The level of SIRT1 was significantly higher in the COT group than in all other groups (*p* < 0.05). The level of PGC-1*α* was significantly higher in the COT and HFT groups than in the HF group (*p* < 0.05).

### 3.3. Meteorin-Like Protein Level in Muscle and Peripheral Tissues

**Figure 4:**
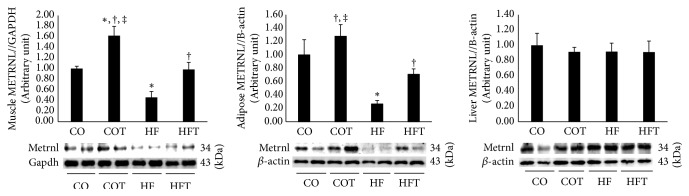
Metrnl protein levels in peripheral tissues following an eight-week training programme. Data are expressed as mean ± SE. CO: normal-diet group, COT: normal-diet + training group, HF: high-fat diet group, and HFT: high-fat diet + training group. *∗* versus CO, *p* < 0.05; † versus HF, *p* < 0.05; ‡ versus HFT, *p* < 0.05.

**Figure 5:**
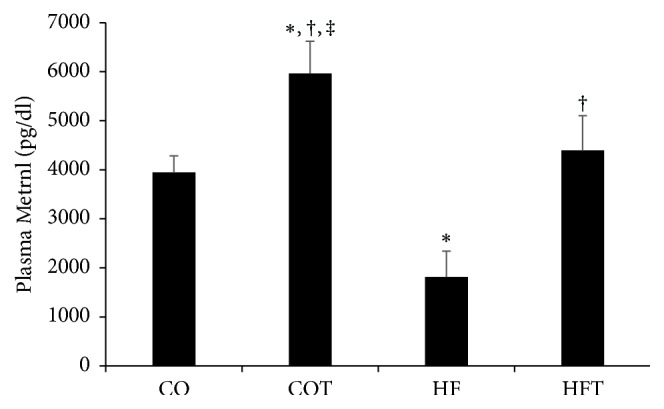
Changes in plasma Metrnl protein levels following an eight-week training programme. Data are expressed as mean ± SE. CO: normal-diet group, COT: normal-diet + training group, HF: high-fat diet group, and HFT: high-fat diet + training group. *∗* versus CO, *p* < 0.05; † versus HF, *p* < 0.05; ‡ versus HFT, *p* < 0.05.

Similar to muscle energy sensing network protein levels, a chronic HFD induced a decrease in Metrnl protein levels in muscle and adipose tissues. However, following eight weeks of regular training, an increase in Metrnl protein levels was observed (Figure 4). Metrnl in muscle and adipose tissues was significantly lower in the HF group than in the CO group (*p* < 0.05). Additionally, the Metrnl levels in the COT and HFT groups were significantly higher than in each control group (*p* < 0.05). Moreover, eight-week training increased plasma Metrnl protein levels in the training groups (Figure 5). However, no significant difference in Metrnl levels in the liver was observed.

### 3.4. Plasma Lipid Profile

Changes in plasma lipid profiles following the eight-week training are presented Table 2. The lipid profiles were significantly higher in the chronic HFD-induced obesity group, but regular exercise improved the lipid profiles. The HF group had significantly higher TC, TG, HDL-c, and LDL-c than the CO group. However, the HFT group had significantly higher HDL-c and significantly lower LDL-c than the HF group. An effect of exercise on the lipid profile was not observed in the control group because these values were within the normal range.

### 3.5. Lipid Metabolism-Related Factors in Adipose Tissue

**Figure 6:**
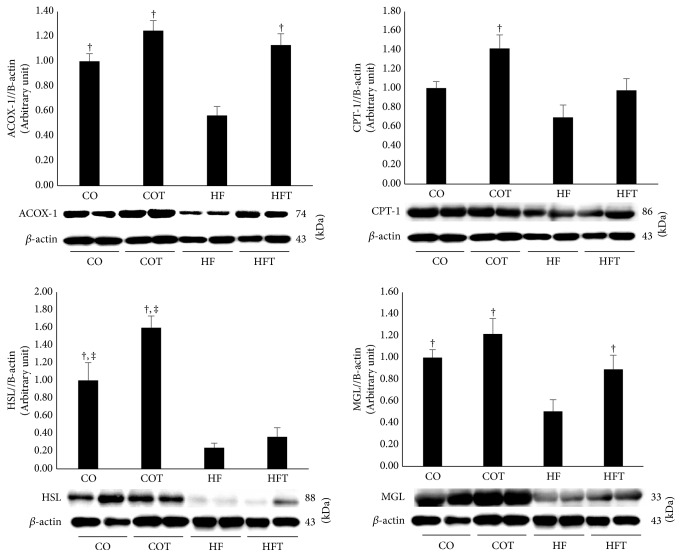
Metabolic makers in adipose tissue following an eight-week training programme. Data are expressed as mean ± SE. CO: normal-diet group, COT: normal-diet + training group, HF: high-fat diet group, and HFT: high-fat diet + training group. *∗* versus CO, *p* < 0.05; † versus HF, *p* < 0.05; ‡ versus HFT, *p* < 0.05.

To determine whether regular exercise improved lipid metabolism-related factors in adipose tissue, *β*-oxidation related factors and lipolysis factors were analysed. Protein levels of all lipid metabolism-related factors in the HF group were significantly decreased, but the HFT group showed improvement (Figure 6). ACOX-1 in adipose tissue was significantly lower in the HF group than in all other groups (*p* < 0.05). HSL was significantly lower in the HF and HFT groups than in the CO and COT groups (*p* < 0.05). MGL was significantly lower in the HF group than in all other groups (*p* < 0.05).

## 4. Discussion

Regular exercise promotes the activity of intramuscular metabolism-related proteins through repeated muscle contraction and relaxation. In the absence of ATP, for example, during fasting and after exercise, energy metabolism is regulated by increasing the level of muscle energy sensing network proteins that lead to AMPK, SIRT1, and PGC-1*α* expression [15, 16]. AMPK is sensitive to changes in the intracellular AMP/ATP ratio and acts to maintain cellular energy stores by switching on catabolic pathways that produce ATP [17, 18]. AMPK enhances SIRT1 activity by increasing cellular NAD^+^ levels [19, 20] and induces SIRT1-mediated deacetylation effects on energy metabolism [20]. Among the muscle energy sensing network proteins, PGC-1*α* plays several roles as a major regulator of energy metabolism. PGC-1*α* is a transcriptional coactivator that increases ATP production in mitochondria and activates nuclear receptors such as peroxisome proliferator-activated receptor *α* (PPAR*α*) and oestrogen-related receptor *α* (ERR*α*) [21]. Increases in muscle energy sensing network proteins have mitigation effects on obesity and Type 2 diabetes by improving energy metabolism [22, 23]. Previous studies reported that muscle energy sensing network proteins are reduced in obesity induced by a 59% HFD [24] and 60% HFD [25]. However, regular exercise increases mitochondrial biogenesis [26] and alleviates insulin resistance and energy metabolism by increasing muscle energy sensing network proteins. Similar to previous studies, the results of this study revealed 24 weeks of an HFD decreased muscle energy sensing network protein levels, but regular aerobic exercise enhanced this protein expression. Notably, the upregulation of PGC-1*α* resulting from an increase in AMPK activity was confirmed by exercise alone without dietary conversion. However, in contrast to previous studies, regular exercise did not induce upregulation of SIRT1 in obese mice. Nevertheless, an increase of PGC-1*α* was observed in this study, which might be a direct consequence of AMPK activation rather than an effect of SIRT1.

Recent studies on exercise-induced changes in muscle protein levels have focused on identifying the transporters that transmit these effects to other tissues. Previous study reported that an increase in intramuscular PGC-1*α* induces the upregulation of Metrnl, which then acts as a messenger responsible for transferring the effect of muscle PGC-1*α* to peripheral tissues. Muscle-specific PGC-1*α* transgenic mice showed an approximately fourfold increased level of muscle Metrnl mRNA and an approximately eightfold increased level in the mass spectrometric analysis from supernatants of cultured myotubes [13]. Regular exercise is thought to increase the level of Metrnl by upregulating the muscle energy sensing network, but its effect has not been determined in the case of HFD-induced obesity. Therefore, it was hypothesised that the protein level of intramuscular Metrnl would change following regular exercise in chronic HFD-induced obese mice. In this study, a decrease in PGC-1*α* and Metrnl protein levels in the muscles of HFD-induced obese mice and an increase in Metrnl in muscle after regular exercise were observed. In a previous study, resistance exercise increased intramuscular Metrnl mRNA levels but did not show any change after aerobic exercise [13]. These conflicting results may be explained by the use of acute aerobic exercise in animals and humans in the previous study.

The mechanisms of transportation and receptor interactions of Metrnl remain unclear. However, previous studies reported evidence that Metrnl correlates with metabolism in peripheral tissues. Previous studies have also shown that plasma Metrnl is increased approximately twofold in conjunction with the increase in intramuscular Metrnl following acute resistance exercise. Moreover, the result of intravenous injections of adenoviral vectors to deliver full-length Metrnl constructs to the liver showed a 20-times increase in liver Metrnl mRNA levels and a 5-6 times increase in plasma three days after administration. Additionally, the increase in Metrnl in adipose tissue increases *β*-oxidation-related mRNA levels including acyl-CoA Synthetase 1 (ACSL-1), ACOX-1, and CPT-1. Increased Metrnl in adipose also increases the levels of uncoupling protein 1 (UCP-1) and type II iodothyronine deiodinase (DIO2), which are brown fat-specific proteins of epididymal and subcutaneous adipose tissue [13]. A previous study also reported that an increase of Metrnl in adipose tissue improves insulin sensitivity through upregulation of PPAR-*γ* [11].

Because in vitro experiments were not conducted in this study, it was not confirmed whether the increase of Metrnl protein in adipose tissue directly affected lipid metabolism. However, previous studies showed that the increase of Metrnl affects lipid oxidation by upregulating *β*-oxidation and brown fat related factors. In this study, Metrnl protein levels in plasma and adipose tissue increased due to regular exercise. Similarly, we confirmed decreased abdominal visceral fat mass and increased *β*-oxidation and lipolysis factors. Therefore, even in a chronically obese state due to a HFD, regular exercise appears to be effective for lipid metabolism in adipose tissue through the increase of Metrnl, which is potentially effective for the treatment of obesity.

## 5. Conclusion

Upregulated muscle energy sensing network proteins caused by regular exercise enhanced intramuscular Metrnl protein levels, resulting in an increase in adipose tissue Metrnl even in a chronically obese state. This study suggests that exercise-induced muscle Metrnl is effective in reducing fat accumulation through the increase of Metrnl in adipose tissue, which may be a therapeutic target for chronic obesity.

## Figures and Tables

**Table 1:** Exercise program.

Duration		Intensity		Time	Frequency
		Speed (m/min)	Slope (°)	(Min)	(Days/week)
Gradually increased	1 week	5	0	5	5
		8	0	10	
		5	0	5	
	2–4 weeks	5	0	10	5
		12	0	30	
		5	0	10	

Equivalent	5–8 weeks	5	0	10	5
		14	0	30	
		5	0	10	

**Table 2:** Data are expressed as mean ± SE. CO: normal-diet group, COT: normal-diet + training group, HF: high-fat diet group, and HFT: high-fat diet + training group. *∗* versus CO, *p* < 0.05; # versus COT, *p* < 0.05; † versus HF, *p* < 0.05.

Variable	CO	COT	HF	HFT
TC (mg/dl)	162.87 ± 8.56	162.09 ± 8.53	301.60 ± 22.06^*∗*,#^	273.56 ± 11.81^*∗*,#^
TG (mg/dl)	62.07 ± 3.43	66.37 ± 3.97	101.40 ± 9.57^*∗*,#^	91.01 ± 7.06^*∗*,#^
HDL-c (mg/dl)	61.21 ± 1.53	72.30 ± 4.61	79.84 ± 8.45^*∗*^	98.84 ± 5.62^*∗*,#,†^
LDL-c (mg/dl)	89.25 ± 6.71	76.52 ± 6.83	201.49 ± 19.48^*∗*,#^	156.51 ± 14.27^*∗*,#,†^
